# Profile of female sex workers suffering from violence in Ukraine

**DOI:** 10.1186/1471-2334-14-S2-P21

**Published:** 2014-05-23

**Authors:** Anna Tokar, Liudmyla Shulga

**Affiliations:** 1 International HIV/AIDS Alliance in Ukraine, Kyiv, Ukraine

## Introduction (aim)

Science 2008 the main rode of HIV transmission began to shift from parenteral toward sexual. So, female-sex workers and FSW- drug users are considered to have increased risk of HIV infection due many factors, some of which could be consequences of personal violence towards females: economic pressure, stigmatized needle-borrowing, sharing of drug preparation equipment and sexual behavior (Choi, 2006; Evans, 2003; Bennett, 2000; Montgomery, 2002). The goal of our research was to define socio-demographic factors associated with risk of personal violence among FSWs.

## Methods

Secondary data analysis was performed on the dataset of bio-behavioral survey with 5023 respondents conducted in 2011. RDS and TLS sampling methodology were applied, only females were recruited. The study was approved by Ethical Review Board of Ukrainian Sociological Association and Institute of epidemiology and infectious diseases named after L. V. Gromashevskyi.

To reveal relationships between ever in life experience of violence, and socio-demographic characteristics of CSWs, binary logistic regression analysis was provided in SPSS 15.0.

## Results

About 55% of respondents were aged 20-30 years old, 52% - completed secondary, vocational school or obtained not full high education, 64% - were unmarried and did not live together with sexual partner. FSWs, who obtained full secondary (OR=1.6 (1.2-2.2) and not full secondary (OR=2.1 (1.4-3.2) education and FSWs of older age group (OR=2.8(2.2-3.6) suffered more often from violence. Those who mostly worked in unsafe environment: on a road (OR=1.2 (1.1-1.4), on a street (OR=2.0 (1.7-2.4), in a hotel (OR=1.9 (1.5-2.4), at a railway station (OR=1.1 (1.0-1.4) – had higher risk to experience violence. Alcohol use during 30 days period (OR=3.7(3.0-4.6) and current drug use (OR=2.2(1.7-2.9) were positively associated with risk of violence.

## Conclusions

Older, less educated CSWs, who provide sexual services in unsafe environment and used alcohol and drugs, had greater risk to experience violence.

